# Bayesian Inference from Count Data Using Discrete Uniform Priors

**DOI:** 10.1371/journal.pone.0074388

**Published:** 2013-10-07

**Authors:** Federico Comoglio, Letizia Fracchia, Maurizio Rinaldi

**Affiliations:** 1 Department of Biosystems Science and Engineering, Swiss Federal Institute of Technology Zürich, Basel, Switzerland; 2 Dipartimento di Scienze del Farmaco, Università degli Studi del Piemonte Orientale “Amedeo Avogadro”, Novara, Italy; Institution and Department: Agricultural Research Service, United States of America

## Abstract

We consider a set of sample counts obtained by sampling arbitrary fractions of a finite volume containing an homogeneously dispersed population of identical objects. We report a Bayesian derivation of the posterior probability distribution of the population size using a binomial likelihood and non-conjugate, discrete uniform priors under sampling with or without replacement. Our derivation yields a computationally feasible formula that can prove useful in a variety of statistical problems involving absolute quantification under uncertainty. We implemented our algorithm in the R package dupiR and compared it with a previously proposed Bayesian method based on a Gamma prior. As a showcase, we demonstrate that our inference framework can be used to estimate bacterial survival curves from measurements characterized by extremely low or zero counts and rather high sampling fractions. All in all, we provide a versatile, general purpose algorithm to infer population sizes from count data, which can find application in a broad spectrum of biological and physical problems.

## Introduction

Absolute quantification of objects, namely the determination of their total number from measurements subject to sampling uncertainty, is a classical problem in statistical inference. In this work, we consider a finite population of identical objects homogeneously dispersed in a finite volume. We assume that measurable fractions of the volume can be sampled and that the number of objects therein can be counted. Given the resulting set of measurements, we address the problem of estimating the population size and its uncertainty using a Bayesian approach with least informative prior distribution.

In a Bayesian treatment of this problem, counts are usually considered to be either Poisson, binomial or negative binomial distributed, depending on the nature of the problem at hand. For example in genomics, over-dispersed sequence count data as those obtained by RNA-Seq are more effectively modeled by a negative binomial than by a Poisson distribution, as the former provides a more flexible mean-variance relationship [1]–[3]. When counts are modeled as a binomial distribution, the binomial likelihood is generally coupled to a conjugate prior to yield a closed form posterior distribution, which corresponds to a simple update of the prior parameters. However, handy computations do not imply that the prior distribution correctly encodes our prior belief, which instead requires specification of both the class of prior distributions and parameters. This choice is paramount when dealing with limited sample sizes [4]–[6], which typically affect biologically relevant inference processes. In addition, in many applications we often have no ground to expect certain simple events to be more likely to occur than others. Therefore, as there is no reason to prefer one distribution over another, a uniform prior distribution can be used to encode this prior belief. This is a formulation of the so called principle of indifference [7], also known as Laplace's principle of insufficient reason [8]. Here, we resort to this principle in order to propose a Bayesian approach in which we introduce the least prior information over a discrete sample space. As the principle of indifference considers each possible outcome as equiprobable, it naturally leads to discrete uniform priors, a class of maximum entropy priors on a discrete sample space [9]–[11]. However, in order to make use of this class of prior distributions for Bayesian inference, we had to address two specific issues: i) a discrete uniform prior with infinite support is an improper prior and ii) it is not a conjugate prior for neither of the above mentioned likelihoods for counts data. Although improper priors are argument of long-standing debate in the field, Jaynes [10] provided a rigorous advice on how to use improper prior for Bayesian inference. Therefore, we addressed the first issue by following Jaynes's approach [10], namely we considered a well defined limit of discrete uniform priors and verified that, even in the limit, the resulting posterior is a proper probability distribution.

Next, despite non-conjugacy we were able to obtain a computationally tractable formula for the posterior distribution of the population size using a binomial likelihood. Particularly, we analyzed two different sampling schemes where objects are either drawn with or without replacement and report a formula for the posterior distribution for each of these cases.

We implemented our algorithms in the R package dupiR and as a showcase, we applied our framework to microbial count data obtained through viable plate counts. A number of studies in clinical and environmental microbiology, and food safety, deal with the quantitative determination of bacteria. Interestingly, low bacterial loads in a sample can challenge bacteria enumeration methods because irrespective of the sampling fraction, they result in low viable counts that are generally considered to be statistically unreliable and hence discarded. By analyzing bacteria survival data exhibiting extremely low counts and rather high sampling fractions, we show that our approach is able to cope well with these data, providing reliable credible intervals for the total number of bacteria even in such extreme cases.

## Results

### General concepts and notation

**Figure 1:**
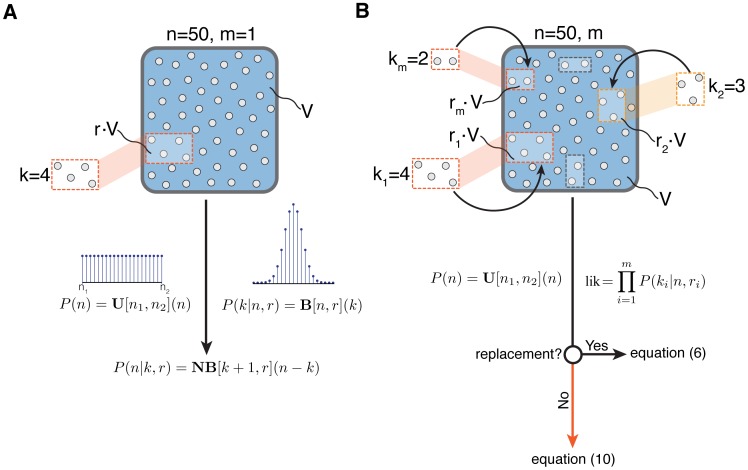
Schematic representation of the problem and of our inference framework. (A) A total of *n* identical objects (in gray, *n* = 50 in this example) is homogeneously dispersed in a finite volume *V*. A fraction *r* of *V*, having volume *rV*, is sampled (dashed red rectangle) and the number of object therein, denoted with *k* (*k* = 4 in this example) is determined. Given the measurement, the posterior distribution of *n* is a negative binomial probability distribution 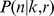 (bottom) computed from a binomial likelihood 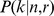 (right) and a discrete uniform prior *P*(*n*) (left). (B) Generalization of (A) to *m* measurements. Fractions of volume 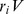 are sampled the number of objects therein (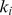) determined as before. However, when *m*>1 two cases can be distinguished: i) the fractions are replaced (sampling with replacement); ii) the fractions are removed from *V* (sampling without replacement). In both cases, we derived a formula for the posterior distribution which is reported in the text as equation 6 for case i and equation 10 for case ii.

We consider a finite volume *V* containing *n* identical and uniformly distributed objects. A single count of *k* objects from a sampling fraction *r*, with 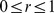, is initially considered (Figure 1A). Our goal is to estimate *n* using a class of discrete uniform priors. Here, counts follow a binomial distribution 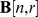


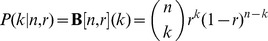
and by Bayes' rule
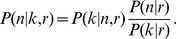
We assume that our prior belief on *n* does not depend on *r*, namely 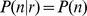, and that *P*(*n*) is the discrete uniform distribution with support 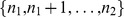 given by
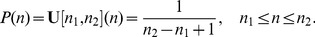
(1)In the following, we consider the general case in which we are given *m* measurements 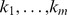 from sampling fractions 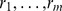 (Figure 1B) and derive a formula for the posterior distribution 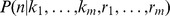 distinguishing between two sampling schemes: i) sampling with replacement; ii) sampling without replacement.

### Derivation of the posterior distribution under sampling with replacement

Here, we derive 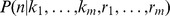 given sample counts drawn with replacement. Assuming *n* to be conditionally independent of 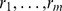, from Bayes' rule we have
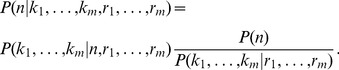
(2)Assuming that the measurements are independent of each other and that counts are conditionally independent of the sample fractions the likelihood factorizes to 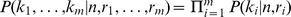 and therefore equation 2 can be written as:
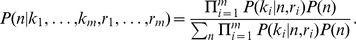
Let 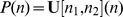 as introduced in equation 1. Then
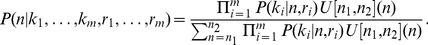
(3)As the interval 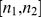 can be arbitrarily large, the denominator of equation 3:
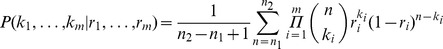
features a potentially intractable summation over the prior support. To address this issue we introduce the following lemma.

**Lemma 1.** Let 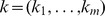, and 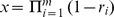. For 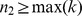


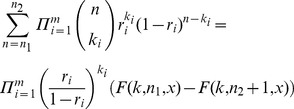
(4)where
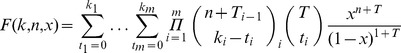
(5)and 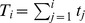, 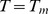, and 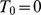. The proof is provided in the Appendix (see Text S1). Based on the fact that the sample counts are generally orders of magnitude smaller than the population size, this lemma allows to replace the sum over *n* by nested sums over 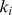, with 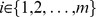. Although the computational complexity of equation 4 is 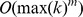, the number of measurements is typically limited in a number of practical applications, thus enabling direct computation of the expression.

Using Lemma 1 we can express the posterior distribution 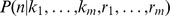 as follows.

**Theorem 1.** If 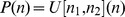 then,
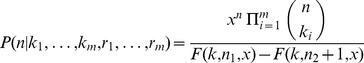
(6)where *k*,*x* are defined as in Lemma 1.

**Proof.** The proof follows by rewriting the posterior distribution of *n* (equation 3) as
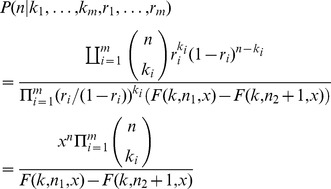



**Corollary 1.** Suppose 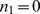. Then 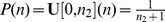, for 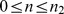, and we have
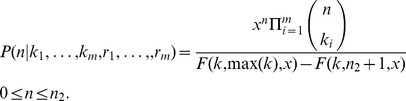



**Corollary 2.** If 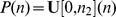 for 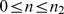 then in the limit 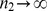


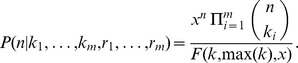
Notice that if a single measurement is given (*m* = 1), the posterior probability of *n* reduces to
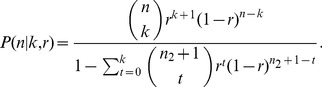
(7)(see Appendix in Text S1). Let *n* = *j*+*k*. In the limit 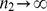 we obtain
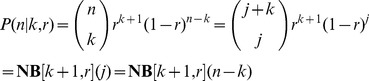
(8)namely, the posterior 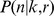 is a negative binomial distribution shifted by *k* units and parametrized by *k*+1 and *r*.

### Derivation of the posterior distribution under sampling without replacement

Suppose that *m* fractions of the volume *V* are sampled uniformly at random without replacement. Let 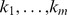 be ordered sample counts, drawn from sampling fractions 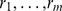 computed with respect to *V*. Clearly, if *m* = 1 the posterior 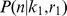 is given by equation 7 and by working in the limit 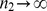 (equation 8) we have
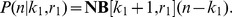
Consider now a second measurement sampled from a fraction 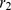 of *V* and therefore equal to a fraction 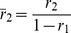 of the residual volume 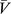. In this case, the likelihood is given by 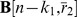 and the prior is 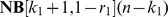. Therefore, the posterior distribution of *n* is given by
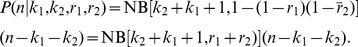
(9)Let 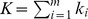 and 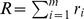. By induction, equation 9 can be generalized to *m* measurements, obtaining
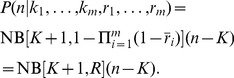
(10)This result has two important properties. First, the computation of the posterior distribution depends only on the sum of the counts and on the sum of the sampling fractions, becoming therefore independent on the number of measurements. As a consequence, any permutation of counts and fractions leads to the same posterior distribution. Second, there exists an equivalence between experiments yielding the same values of *K* and *R* through a different number of measurements, i.e. counting 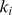 in fractions 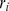 for 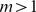 yields the same posterior distribution as counting 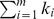 counts in a fraction 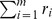 in single measurement.

### The R package dupiR

We implemented our algorithms as a package for the statistical environment R [12]. The package, which we called dupiR (discrete uniform prior-based inference with R) is available from the Comprehensive R Achive Network (CRAN) along with the relevant package manual. dupiR is based on the custom S4 class Counts, which is used to store sample information, statistical attributes and inference results. By default, the package assumes that samples have been drawn without replacement. Given a set of sample counts 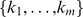 and fractions 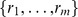, dupiR defines the default support interval for the discrete uniform prior distribution as 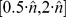, where 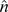 is the maximum likelihood estimate of *n* computed as 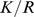, where 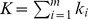 and 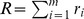. For the special case *K* = 0, the prior support is defined as 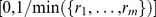. This setup proved to be effective across a variety of simulated measurements. However, the user can override default values by explicitly using the variables n1 and n2 to define a custom prior support.

Posterior distributions can be computed using the function computePosterior, where the logical parameter replacement specifies whether counts were sampled with or without replacement. Posterior parameters can be obtained using getPosteriorParam, which returns a point estimate of *n* equal to its maximum a posteriori (MAP) and the corresponding credible interval at a specified confidence level (default to 95%), among other parameters. Finally, dupiR can be used to produce publication-level quality figures representing posterior distributions and parameters simply via the plot function. Further information are provided in the package documentation.

### Applications to bacterial enumeration

Absolute quantification of bacteria in biological samples is performed routinely for a broad spectrum of applications ranging from diagnostics to food analysis. A standard method for bacterial enumeration is the plate count method, which despite well-recognized limitations provides an indirect measure of cell density solely based on viable bacteria [13]. Viable plate counts - the discrete outcome of this method - are then generally used to compute point estimates of the bacterial concentration in the original sample. Although Bayesian estimates of the uncertainty associated to bacteria quantification have been previously proposed, these methods assume Poisson distributed microbial counts [14], [15]. Particularly, Clough *et al.* [14] adopted a Poisson likelihood 
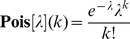
 with rate *λ* = *rn* and a Gamma prior distribution 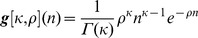, where *κ* and *ρ* are the shape and the rate parameters, respectively. It then follows that the posterior distribution 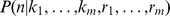 is itself a Gamma distribution given by
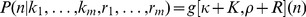
(11)where 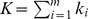 and 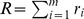. Hereinafter, we will refer to this setup as the GP (Gamma-Poisson) method. In applying the GP method to bacteria enumeration, the authors chose *κ* = 1 and 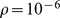. Notice that the gamma distribution is appropriate to model continuous variables and therefore a continuous approximation to *n* is assumed in this model.

By analyzing equation 11 we can observe that when 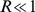 and 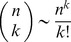, the expression converges to our posterior distribution under sampling without replacement (equation 10) as
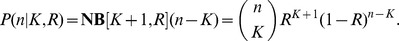
Indeed notice that for small values of *R* the expression above depends on *n* as 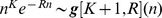 and that by setting *ρ* = 0, 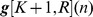 is equal to equation 11.

**Figure 2:**
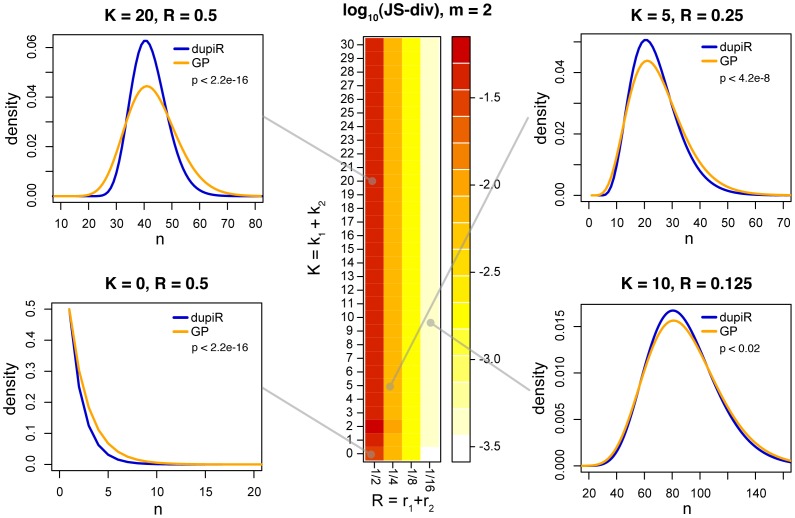
Comparison between posterior distributions computed with dupiR and with the GP method. Middle: JS-divergence (expressed in log_10_) between posterior distributions computed with our algorithm using sampling without replacement or with the GP method (Clough *et al.* [14]) as a function of total counts (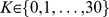, see Methods) and total sampling fractions (*R*) obtained from two measurements (*m* = 2). Right and Left: examples of posterior distributions corresponding to values of (*K*,*R*) indicated by grey lines are illustrated. *p*-values have been computed using a two-sided Kolmogorov-Smirnov test.

Convergence implies that for a broad range of measurements our inference framework and the GP method provide comparable results. However, when the difference between sampling methods is not negligible, i.e. when sampling fractions are large, the results provided by the two methods become significantly different. To investigate this difference in greater details and to assess the performances of our inference framework, we simulated measurements from total sampling fractions spanning two orders of magnitude and we compared the posterior distributions inferred with our method to those obtained via the GP method using the Jensen-Shannon divergence (JS-divergence), a symmetric version of the Kullback-Leibler divergence (see Methods). Our simulation results show that when *R* is so small that the effect of replacement is negligible, posterior distributions computed using our method or with the GP method correspond to the same probability distribution for any practical purpose (Figure S1). More precisely, the effect of replacement can be neglected when 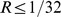, a value at which the JS-divergence between posterior distributions computed from sampling with and without replacement drops below 10^−4^ (Figure S2). However, when *R*>1/32, the two approaches differ substantially. For these values of total sampling fractions, posterior distributions computed using our algorithm exhibit a lower variance than those computed with the GP method (Figure 2), thus providing narrower credible intervals. It is noteworthy to observe that this result is not a mere consequence of an inappropriate parametrization of the Gamma prior. Indeed, simulations performed by varying *ρ* over several orders of magnitude (from 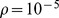 to *ρ* = 0.1) showed that differences between posterior distributions remain significant irrespective of *ρ* (Figure S3, A–E). Rather, as expected, extreme rate parameters can lead to posterior distributions that are dominated by prior belief (see Figure S3, F for an example), emphasizing the importance of an appropriate prior parametrization.

**Figure 3:**
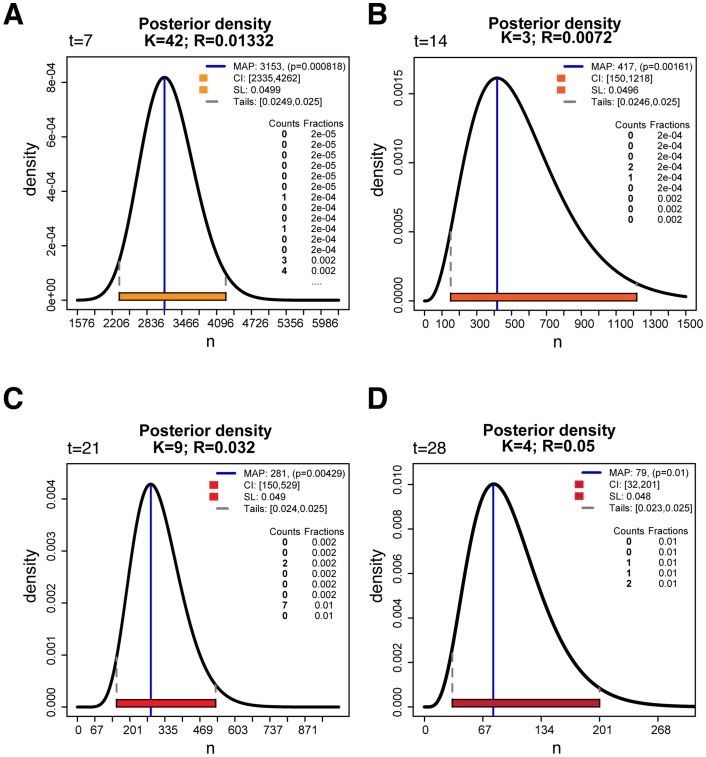
Examples of dupiR graphical output. Examples of posterior distributions of the population size *n* estimated and plotted with dupiR for time points (A) *t* = 7, (B) *t* = 14 (C) *t* = 21 and (D) *t* = 28 days. By default, the graph of the posterior distribution (solid black line) is plotted along with a statistical summary containing the maximum a posteriori (MAP, indicated by the blue vertical line) of *n*, the corresponding credible intervals (CI, green and dashed grey lines) at a significance level (SL) of 0.05 and the tails probability of the distribution function.

**Figure 4:**
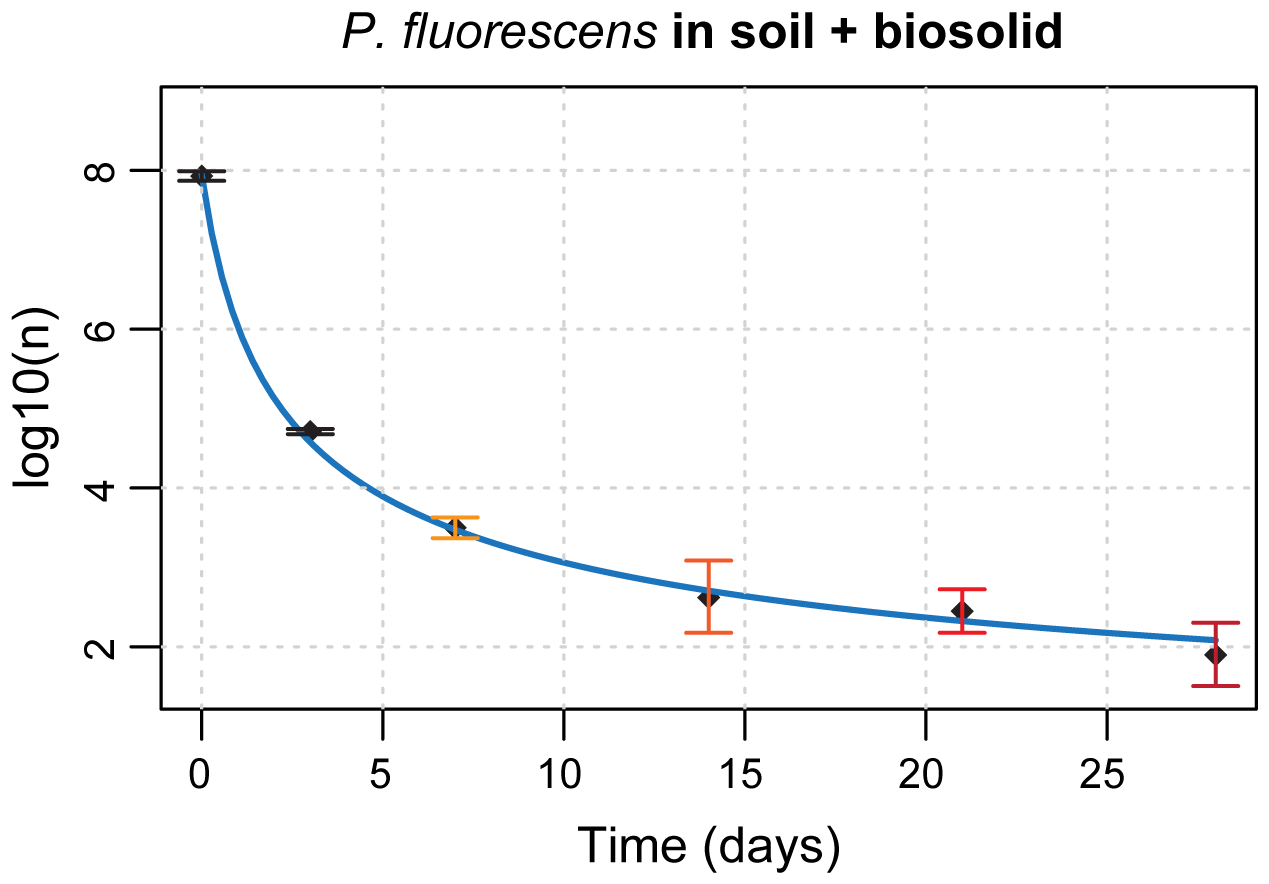
Application of discrete uniform priors to bacterial survival curves estimation. Estimated bacterial survival curve (light blue line, see Methods) of *P. fluorescens* inoculated in soil supplemented with biosolid. Time points from *t* = 7 to *t* = 28, characterized by zero or extremely low viable counts, are indicated in tones of red and the corresponding posterior distributions are shown in Figure 3.

Taken together, these results underscore the generality of our inference method, which is able to cope with measurements derived from any range of *K* and *R*, including extreme total sampling fractions and counts. This latter property is desirable for bacterial enumeration. In fact, only measurements with 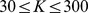 are routinely used to infer the population size [16] and those localizing outside this range are currently discarded. Clearly, if *K*<30 and 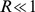 it is often easy to obtain a measurement with *K* falling within the recommended range simply by considering those samples in the dilution series which are less diluted (i.e. obtained from a higher sampling fraction). However, when *n* is small, measurements obtained from high sampling fractions can still yield low counts. Studies investigating bacterial survival upon physical or chemical treatments or in different environmental conditions are often confronted with this limitation. Bacterial survival studies are generally based on time-course bacterial enumeration using different experimental techniques and aim to estimate bacterial survival curves that in turn are used to compare cell viability across conditions. In a recent environmental microbiology study, Fracchia *et al.* investigated the suitability of biosolids as inoculum vehicle for the plant-growth promoting rhizobacteria *Pseudomonas fluorescens* [17]. Here, we deal with a single time series which was generated as described in [17] (six time points where for each sample at least five technical replicates were subjected to bacterial enumeration, see Methods) where only the first two time points yielded 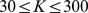 and where more than 50% of the measurements in later time points showed *K* = 0 (see Figure 3). Instead of discarding these measurements, we applied dupiR to compute posterior distributions and estimated the maximum a posteriori (MAP) of *n* from all time points. These values were then used to fit a power-law model (see Methods) that shows good agreement with the experimental data (residual standard error of 0.1269 on 3 degrees of freedom), thus enabling us to estimate a survival curve of *P. fluorescens* in a time series characterized by extremely low viable counts (Figure 4). Clearly, dupiR estimates can be integrated into more complex models of cell growth or survival for which several mathematical approaches have been proposed [18]–[21].

## Discussion

Parametrization of the prior probability distribution is a key step in Bayesian statistics. This step requires particular care for small sample sizes, as posterior distributions can be easily dominated by prior belief unless the parameters reflect an appropriate equivalent sample size of the prior distribution [4], [5]. In addition, the choice of the prior is sometimes driven by convenience rather than prior belief. In this study, we set out to overcome these intrinsic limitations by implementing Keynes' principle of indifference [7] in a Bayesian framework to infer population sizes (*n*) from sample measurements. Notably, we did not limit ourselves to a theoretical treatment of the subject but we provide an optimized, general purpose implementation of our algorithm in the R package dupiR.

By attributing equal probabilities to each possible outcome, Keynes' principle of indifference is naturally encoded in discrete uniform priors. Notice that the application of this principle in our univariate, discrete problem is free from possible unexpected behavior that are known to arise in multivariate, continuous applications (e.g. see [22]). Although discrete uniform priors are not conjugate for likelihoods commonly adopted in dealing with count data, we were able to derive the posterior probability distribution of *n* using a binomial likelihood. If data are obtained through sampling with replacement, we report a computationally tractable formula of the posterior distribution of *n* which could be obtained by converting a summation over the prior support to multiple summations over the range of sample counts only. Indeed, while the former can be theoretically unbound, sample counts are typically orders of magnitude smaller than *n*. The special case in which only a single measurement is available leads to a negative binomial posterior distribution, which was then used as a building block to extend our framework to an arbitrary number of measurements obtained from sampling without replacement. The properties of the posterior distributions we obtained depend on the sampling method. Particularly, while under sampling with replacement measurements contribute individually to the inference process, if no replacement is performed then the posterior distribution depends only on the total of sample counts and fractions. This property allows computations to be independent of the number of measurements.

The sampling method has no influence on the result if the total sampling fraction is modest compared to the total volume (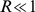). This holds true for typical experimental settings and under this condition the performances of our algorithm are comparable to those of other Bayesian methods reported in literature, such as the GP method [14] (Figures S1 and S2). However, the results of the two methods diverge when the effect of replacement can no longer be neglected. In this cases, our method provides posterior distributions that are characterized by a significantly smaller variance compared to those obtained using a Gamma prior (Figure 2). This property can be seen analytically. Since 
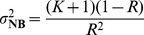
 and 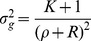, when 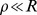 we have 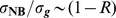 and hence 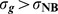.

We showed an application of our method in the context of bacterial enumeration, where we investigated the survival of an engineered strain of *P. fluorescens* using a time series with very low or zero viable counts and rather high sampling fractions. Although in this work we dealt with viable plate counts only, data generated by other laboratory techniques, such as the direct count [23] and the drop plate method [24] can be analyzed with dupiR. In addition, combining our algorithm with automatic plate counting [25] could result in a reliable and robust pipeline for bacteria enumeration via plate counting methods.

All in all, we provided a general purpose algorithm to infer population sizes from count data. We believe that the method can be applied to a broad spectrum of applications in both biological and physical sciences.

## Materials and Methods

### Simulation

Given a set of total counts *K* and a set of total sampling fractions *R* we considered the pairs *K*×*R* and computed posterior distributions using either our posterior formula under sampling without replacement or the GP method. For each 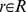, all posterior distributions were computed using the same discrete uniform prior by setting its support to the interval 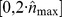, where 
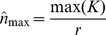
. Posterior distributions computed via the two methods were compared by computing the Jensen-Shannon divergence (JS-divergence), a symmetrised Kullback-Leibler divergence (KL-divergence) [26]. Given two discrete probability distributions *p* and *q*, the KL-divergence (in bits) is defined as
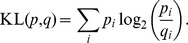
Since the KL-divergence is not symmetric, different symmetrization procedures have been proposed in literature. Here we used a symmetric form of the KL-divergence known as Jensen-Shannon divergence (JS-divergence) [27]. By letting 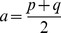 be the average distribution of *p* and *q*, the JS-divergence is defined as
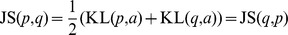
and represents the average KL-divergence of the distributions *p*,*q* to the average distribution *a*. When JS(*p*,*q*) is computed in bits we have 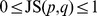. Therefore, in this work we always considered the quantity 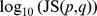.

### Experimental procedure

Viable plate counts of *Pseudomonas fluorescens* were obtained essentially as described in [17] in a single time series (six time points). Briefly, bacteria of the rifampicin and tetracyclin resistant strain *P. fluorescens* 92*^RTcgfp^* carrying the *gfp* gene were precultured to a density of 10^8^–10^9^ cells/ml. The bacterial suspension was then inoculated into a microcosm consisting of soil supplemented with biosolid and incubated at 25 C° in the dark over a period of 28 days. Inoculation corresponds to the time point *t* = 0. Samples were collected at time points *t* = 3,7,14,21 and *t* = 28 days, subjected to log10 serial dilution and plated on LB agar added with rifampicin, tetracycline and cycloheximide. For each time point, viable plate counts were determined from five or more technical replicates.

### Estimation of survival curves

For each time point, posterior distributions were computed using dupiR and sampling without replacement. Survival curves were fit using a power-law model
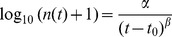
where *n*(*t*) is the maximum a posteriori of the population size at time point *t*. The model was fit using the R function nls [12] and starting estimates 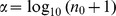, *β* = 0.2 and 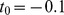.

## Supporting Information

Text S1 **Appendix.** This supplementary file is an Appendix containing the proof of Lemma 1 and additional information pertaining the derivation of the posterior distributions discussed in the main text.
(PDF)

Figure S1
**Simulation results.** JS-divergence (expressed in log_10_) between posterior distributions computed with our method without replacement or with the GP method as a function of the total sampling fractions (*R*). Total counts 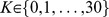 have been considered.(TIF)

Figure S2 **Maximum JS-divergence as a function of the total sampling fraction.** Maximum JS-divergence as a function of *R*. The red line indicates a linear regression fit. The orange vertical dashed line indicates the value *R* = 1/32. For *R*<1/32, posterior distributions computed with or without replacement can be considered to be the same for any practical application.
(TIF)

Figure S3 **Comparison between dupiR and the GP method for different Gamma prior parameters.** JS-divergence (expressed in log_10_) between posterior distributions computed with dupiR and sampling without replacement or with the GP method (Clough *et al.* [14]) as a function of total counts (*K*) and total sampling fractions (*R*) obtained from two measurements (*m* = 2, see Methods). The rate parameter (*ρ*) of the Gamma prior was varied over four orders of magnitude and different panels correspond to simulations run with (A) *ρ* = 10^−5^, (B) *ρ* = 10^−4^, (C) *ρ* = 10^−3^, (D) *ρ* = 10^−2^, (E) *ρ* = 0.1. (F) Example of the effect of the Gamma prior parametrization on the posterior distribution inferred from *K* = 5,*R* = 0.25 and *ρ* = 10^−6^ (orange), *ρ* = 10^−2^ (red) and *ρ* = 0.1 (brown). The latter case encodes a prior of *K* = 1 from *R* = 0.1. The posterior distribution estimated with dupiR is shown in blue, with the maximum a posteriori of *n* indicated by the dashed gray line.
(TIF)
