# Supplementary material for: Bayesian Inference from Count Data Using Discrete Uniform Priors
Source: PLoS One. 2013 Oct 7;8(10):e74388. doi: 10.1371/journal.pone.0074388 (PMC3792115; doi:10.1371/journal.pone.0074388)
Supplement: Text S1 — Appendix. This supplementary file is an Appendix containing the proof of Lemma 1 and additional information pertaining the derivation of the posterior distributions discussed in the main text. (PDF) [file pone.0074388.s001.pdf]

Supporting Information for: Bayesian inference from count data using discrete uniform priors

Federico Comoglio[1,†], Letizia Fracchia[2] and Maurizio Rinaldi *[2,†]

[1]Department of Biosystems Science and Engineering, Swiss Federal Institute of Technology Zürich, Mattenstrasse 26, Basel, Switzerland
[2]Dipartimento di Scienze del Farmaco, Università degli Studi del Piemonte Orientale "Amedeo Avogadro", Largo Donegani 2, Novara, Italy
[†]These authors contributed equally to this work.

June 14, 2013

Contents

1 Appendix **2**
1.1 Proof of Lemma 1 2
1.2 Special case with a single measurement 3

*Correspondence should be addressed to M.R. (maurizio.rinaldi@unipmn.it)

1 Appendix

1.1 Proof of Lemma 1

Lemma 1.1 Let $Z = Z(x,y) = xy$ and $k, s, n \geq 0$. Then

$$D_x^k\left(\frac{Z^n}{(1-Z)^s}\right) = \frac{k!}{x^k}\sum_{t=0}^{k}\binom{n}{k-t}\binom{s+t}{t}\frac{Z^{n+t}}{(1-Z)^{s+t+1}}.$$

where D_x denotes the derivative with respect to the variable x.

Proof By applying the Leibnitz rule we get

$$\begin{aligned}
D_x^k\left(\frac{Z^n}{(1-Z)^{s+1}}\right) &= \sum_{t=0}^{k}\binom{k}{t}D_x^{k-t}(Z^n)D_x^t\left(\frac{1}{(1-Z)^{s+1}}\right)\\
&= \sum_{t=0}^{k}(k-t)!\binom{k}{t}\binom{n}{k-t}Z^{n-k+t}y^{k-t}\frac{(s+t)!}{s!}\frac{y^t}{(1-Z)^{s+t+1}}\\
&= k!\sum_{t=0}^{k}\binom{n}{k-t}\binom{s+t}{t}\frac{Z^{n+t-k}y^k}{(1-Z)^{s+t+1}}\\
&= k!\sum_{t=0}^{k}\binom{n}{k-t}\binom{s+t}{t}\frac{Z^{n+t}}{x^k(1-Z)^{s+t+1}}. \quad \square
\end{aligned}$$

Lemma 1.2 Let $Z_m = Z_m(x_1, \ldots, x_m, y) = x_1 \cdots x_m y$. Then

$$D_{x_1}^{k_1}\cdots D_{x_m}^{k_m}\left(\frac{Z_m^n}{(1-Z_m)^{s+1}}\right) = \prod_{i=1}^{m}\frac{k_i!}{x_i^{k_i}}\sum_{t_1=0}^{k_1}\cdots\sum_{t_m=0}^{k_m}\prod_{i=1}^{m}\binom{n+T_{i-1}}{k_i-t_i}\binom{s+T_i}{t_i}\frac{Z_m^{n+T}}{(1-Z_m)^{s+T+1}}, \tag{1}$$

where $T_s = \sum_{j=1}^{s} t_j$, $T = T_m$ and $T_0 = 0$.

Proof The proof follows by induction on m. The assertion holds for $m = 1$ where it reduces to the statement of Lemma 1.1. Next, let us suppose that the assertion (1) holds for m and let us prove it for $m+1$. Notice that $Z_{m+1}(x_1, \ldots, x_{m+1}, y) = x_1 \cdots x_{m+1}y = Z_m(x_1, \ldots, x_m, yx_{m+1})$. We then get

$$\begin{aligned}
&D_{x_1}^{k_1}\cdots D_{x_m}^{k_m}D_{x_{m+1}}^{k_{m+1}}\left(\frac{Z_m(x_1,\ldots,x_m,yx_{m+1})^n}{(1-Z_m(x_1,\ldots,x_m,yx_{m+1}))^s}\right)\\
&= D_{x_{m+1}}^{k_{m+1}}\prod_{i=1}^{m}\frac{k_i!}{x_i^{k_i}}\sum_{t_1=0}^{k_1}\cdots\sum_{t_m=0}^{k_m}\prod_{i=1}^{m}\binom{n+T_{i-1}}{k_i-t_i}\binom{s+T_i}{t_i}\frac{Z_m(x_1,\ldots,x_m,yx_{m+1})^{n+T_m}}{(1-Z_m(x_1,\ldots,x_m,yx_{m+1}))^{s+T_m+1}}\\
&= \left(\prod_{i=1}^{m}\frac{k_i!}{x_i^{k_i}}\right)\sum_{t_1=0}^{k_1}\cdots\sum_{t_m=0}^{k_m}\prod_{i=1}^{m}\binom{n+T_{i-1}}{k_i-t_i}\binom{s+T_i}{t_i}D_{x_{m+1}}^{k_{m+1}}\frac{Z(x_{m+1},x_1\cdot\ldots\cdot x_m y)^{n+T_m}}{(1-x_{m+1}Z(x_{m+1},x_1\cdot\ldots\cdot x_m y)^{s+T_m+1}}. \quad \square
\end{aligned}$$

By applying again Lemma 1.1 we can write

$$D_{x_1}^{k_1}\cdots D_{x_m}^{k_m}D_{x_{m+1}}^{k_{m+1}}\left(\frac{Z_{m+1}(x_1,\ldots,x_{m+1},y)^n}{(1-Z_{m+1}(x_1,\ldots,x_{m+1},y))^s}\right)=\left(\prod_{i=1}^{m}\frac{k_i!}{x_i^{k_i}}\right)\sum_{t_1=0}^{k_1}\cdots\sum_{t_m=0}^{k_m}\prod_{i=1}^{m}\binom{n+T_{i-1}}{k_i-t_i}\binom{s+T_i}{t_i}\times$$

$$\frac{k_{m+1}!}{x_{m+1}^{k_{m+1}}}\sum_{t_{m+1}=0}^{k_{m+1}}\binom{n+T_m}{k_{m+1}-t_{m+1}}\binom{s+T_m+t_{m+1}}{t_{m+1}}\frac{Z(x_{m+1},x_1\cdot\ldots\cdot x_m y)^{n+T_m+t_{m+1}}}{(1-Z(x_{m+1},x_1\cdot\ldots\cdot x_m y))^{s+t_{m+1}+T_m+1}}$$

$$=\left(\prod_{i=1}^{m+1}\frac{k_i!}{x_i^{k_i}}\right)\sum_{t_1=0}^{k_1}\cdots\sum_{t_{m+1}=0}^{k_{m+1}}\prod_{i=1}^{m+1}\binom{n+T_{i-1}}{k_i-t_i}\binom{s+T_i}{t_i}\frac{Z_{m+1}(x_1,\ldots,x_{m+1},y))^{n+T_{m+1}}}{(1-Z_{m+1}(x_1,\ldots,x_{m+1},y)))^{s+T_{m+1}+1}}.\square$$

Setting $y=1$ and $s=0$ in Lemma 1.2 gives:

Corollary 1.1

$$D_{x_1}^{k_1}\cdots D_{x_m}^{k_m}\left(\frac{(x_1\cdots x_m)^n}{1-(x_1\cdots x_m)}\right)=\prod_{i=1}^{m}\frac{k_i!}{x_i^{k_i}}F(k,n,x_1\cdots x_m)$$

where $k=(k_1,\ldots k_m)$ and

$$F(k,n,x)=\sum_{t_1=0}^{k_1}\cdots\sum_{t_m=0}^{k_m}\prod_{i=1}^{m}\binom{n+T_{i-1}}{k_i-t_i}\binom{T_i}{t_i}\frac{x^{n+T}}{(1-x)^{T+1}}$$

We can now prove Lemma 1. Let $k=(k_1,\ldots,k_m)$, $r=(r_1,\ldots,r_m)$, $x_i=1-r_i$ and $x=\prod_{i=1}^{m}x_i$.

Proof We have

$$\sum_{n=0}^{n_2}\prod_{i=1}^{m}\binom{n}{k_i}r_i^{k_i}(1-r_i)^{n-k_i}=\sum_{n=0}^{n_2}\prod_{i=1}^{m}\frac{r_i^{k_i}}{k_i!}D_{x_i}^{k_i}x_i^n=$$

$$=\prod_{i=1}^{m}\frac{r_i^{k_i}}{k_i!}D_{x_i}^{k_i}\sum_{n=\max(k)}^{n_2}x^n=\prod_{i=1}^{m}\frac{r_i^{k_i}}{k_i!}D_{x_i}^{k_i}\left(\frac{x^{\max(k)}-x^{n_2+1}}{1-x}\right)$$

$$=\prod_{i=1}^{m}\left(\frac{r_i}{1-r_i}\right)^{k_i}\left(F(k,\max(k),x)-F(k,n_2+1,x)\right).\quad\square$$

Notice that

$$\sum_{n=n_1}^{n_2}\prod_{i=1}^{m}\binom{n}{k_i}r_i^{k_i}(1-r_i)^{n-k_i}=\left(\sum_{n=0}^{n_2}-\sum_{n=0}^{n_1-1}\right)\prod_{i=1}^{m}\binom{n}{k_i}r_i^{k_i}(1-r_i)^{n-k_i}$$

$$=\prod_{i=1}^{m}\left(\frac{r_i}{1-r_i}\right)^{k_i}\left(F(k,n_1,x)-F(k,n_2+1,x)\right).$$

As a consequence, we also have $F(k,\max(K),x)=F(k,i,x)$ for $i=0,\ldots,\max(K)$.

1.2 Special case with a single measurement

If $m=1$ (a single measurement) and $n_1=0$, $r=r_1$, $k=k_1$ then equation 6 in the main text becomes

$$
\begin{aligned}
P(n|k,r) &= \frac{\binom{n}{k}(1-r)^n}{\frac{(1-r)^k}{r^{1+k}} - \sum_{t=0}^{k}\binom{n_2+1}{k-t}\frac{(1-r)^{n_2+1+t}}{r^{1+t}}} \\
&= \frac{\binom{n}{k}r(1-r)^n}{\frac{(1-r)^k}{r^k} - \sum_{t=0}^{k}(1-r)^{n_2+1}\binom{n_2+1}{k-t}\frac{(1-r)^t}{r^t}} \\
&= \frac{\binom{n}{k}r^{k+1}(1-r)^{n-k}}{1 - \sum_{t=0}^{k}(1-r)^{n_2+1}\binom{n_2+1}{k-t}\frac{(1-r)^{t-k}}{r^{t-k}}} = \frac{\binom{n}{k}r^{k+1}(1-r)^{n-k}}{1 - \sum_{t=0}^{k}(1-r)^{n_2+1-t}\binom{n_2+1}{t}r^t}
\end{aligned}
$$

thus obtaining equation 7 in the main text.
