# Supplementary figures and images for: Bayesian Inference from Count Data Using Discrete Uniform Priors
Source: PLoS One. 2013 Oct 7;8(10):e74388. doi: 10.1371/journal.pone.0074388 (PMC3792115; doi:10.1371/journal.pone.0074388)

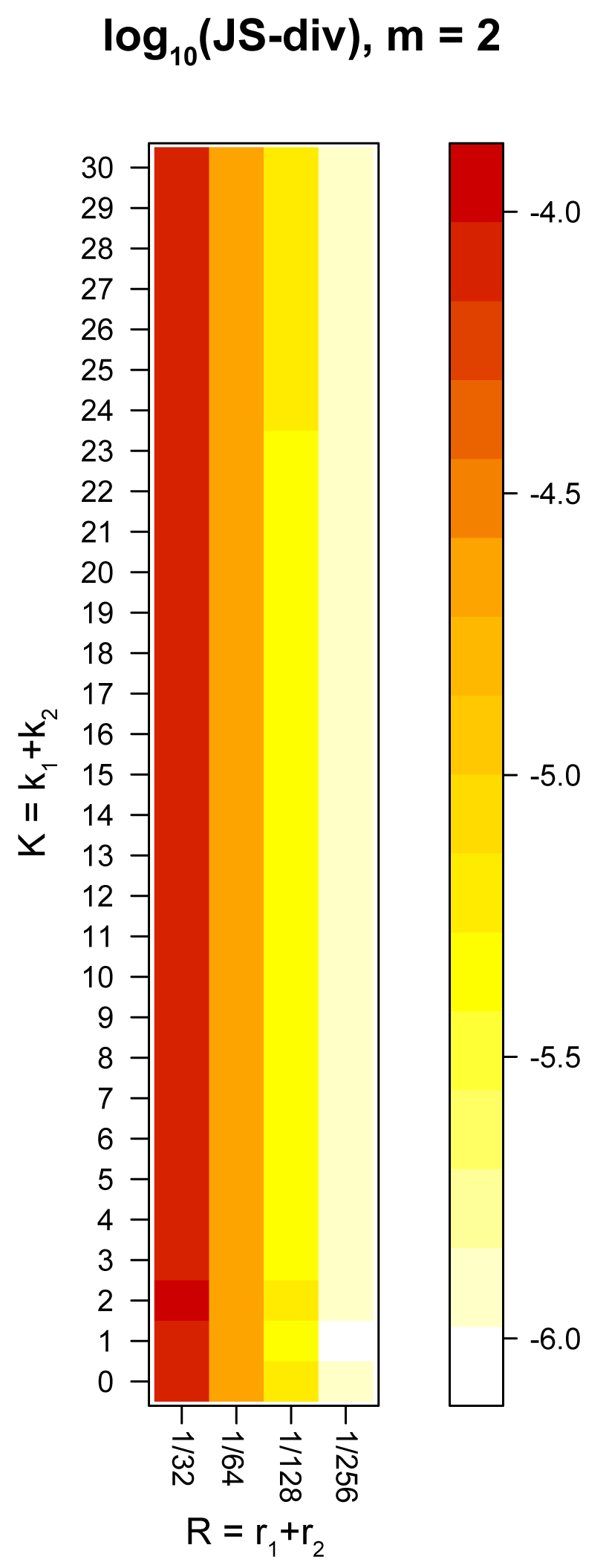

Supplement: Figure S1 — Simulation results. JS-divergence (expressed in log10) between posterior distributions computed with our method without replacement or with the GP method as a function of the total sampling fractions (R). Total counts have been considered. (TIF) [file pone.0074388.s002.tif]

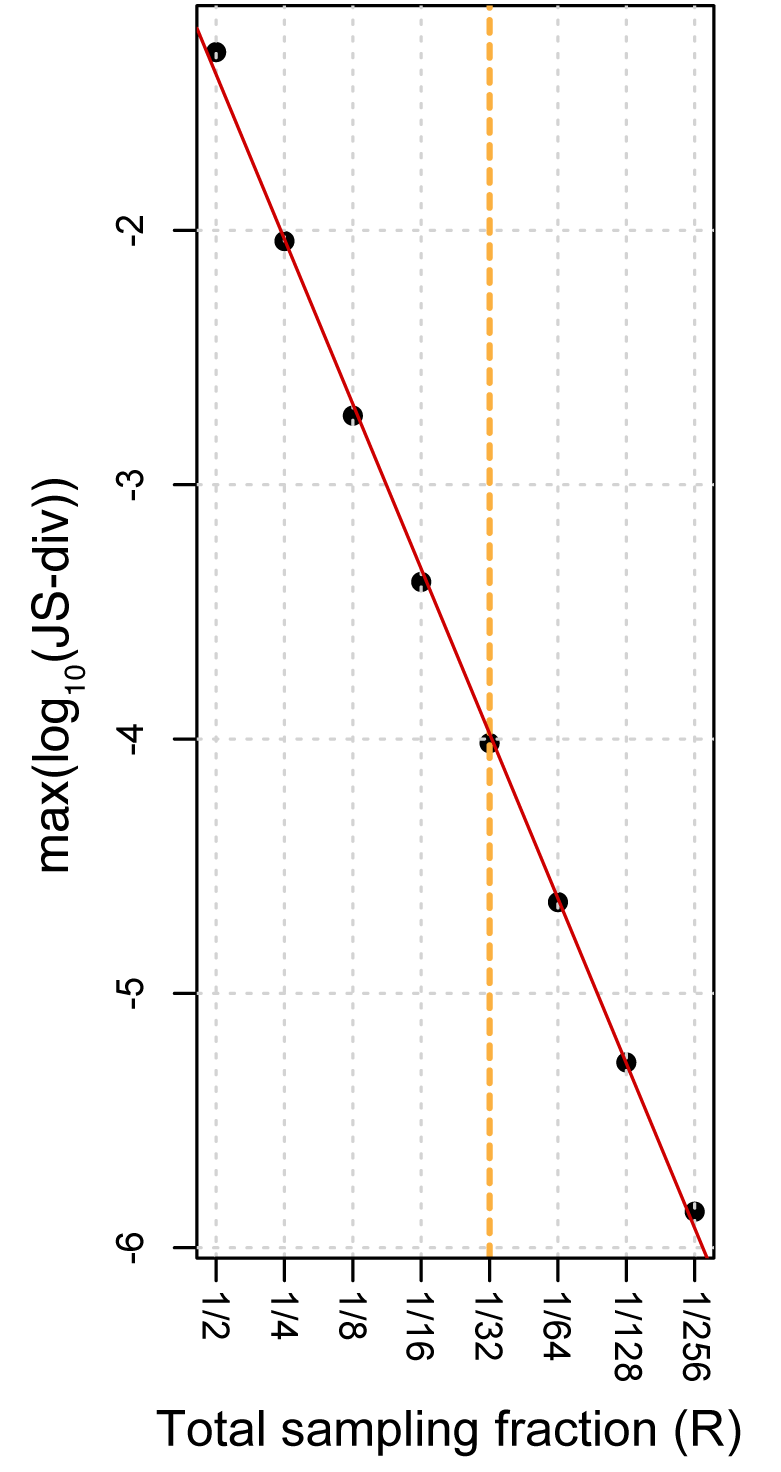

Supplement: Figure S2 — Maximum JS-divergence as a function of the total sampling fraction. Maximum JS-divergence as a function of R. The red line indicates a linear regression fit. The orange vertical dashed line indicates the value R = 1/32. For R<1/32, posterior distributions computed with or without replacement can be considered to be the same for any practical application. (TIF) [file pone.0074388.s003.tif]

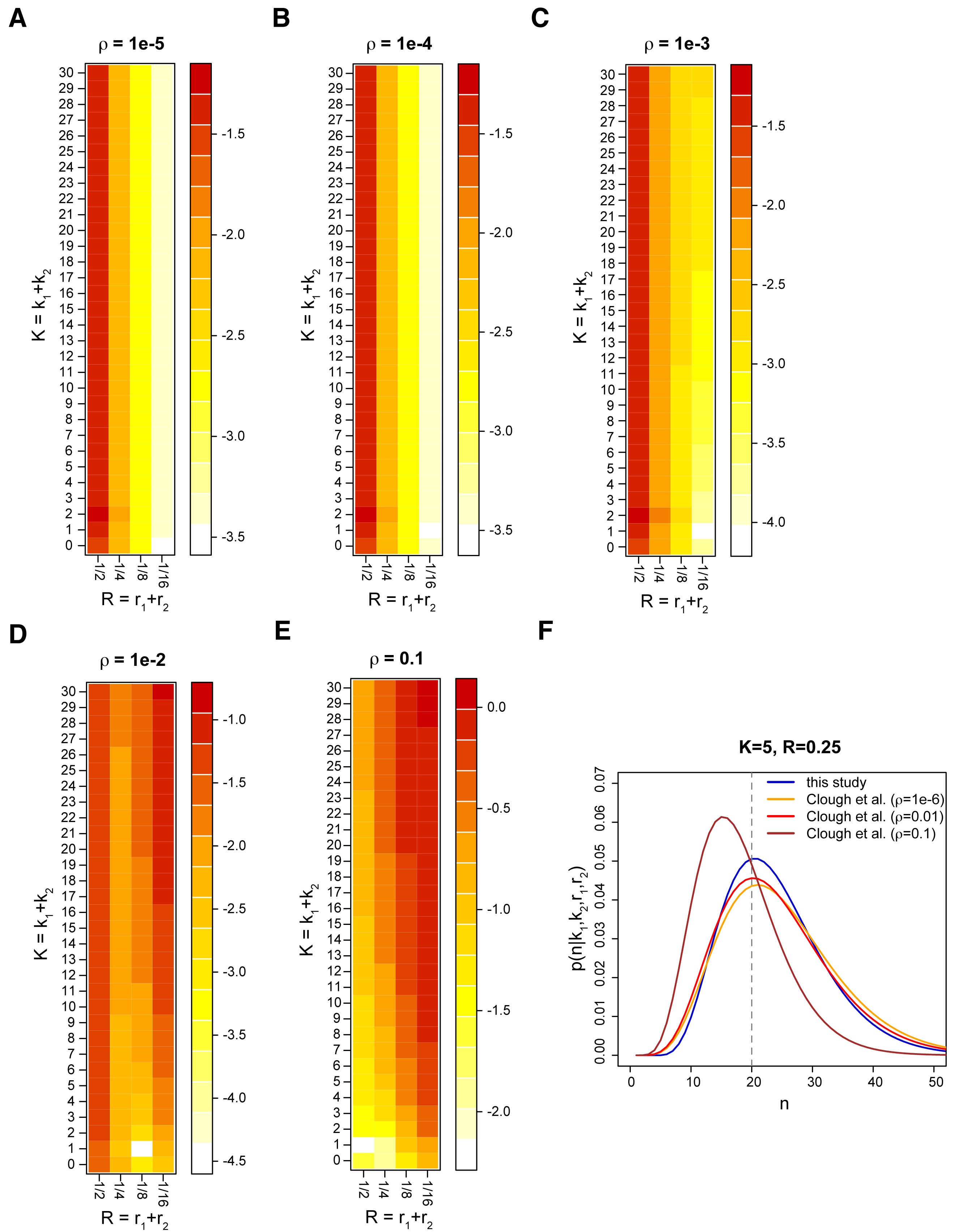

Supplement: Figure S3 — Comparison between dupiR and the GP method for different Gamma prior parameters. JS-divergence (expressed in log10) between posterior distributions computed with dupiR and sampling without replacement or with the GP method (Clough et al. [14]) as a function of total counts (K) and total sampling fractions (R) obtained from two measurements (m = 2, see Methods). The rate parameter (ρ) of the Gamma prior was varied over four orders of magnitude and different panels correspond to simulations run with (A) ρ = 10−5, (B) ρ = 10−4, (C) ρ = 10−3, (D) ρ = 10−2, (E) ρ = 0.1. (F) Example of the effect of the Gamma prior parametrization on the posterior distribution inferred from K = 5,R = 0.25 and ρ = 10−6 (orange), ρ = 10−2 (red) and ρ = 0.1 (brown). The latter case encodes a prior of K = 1 from R = 0.1. The posterior distribution estimated with dupiR is shown in blue, with the maximum a posteriori of n indicated by the dashed gray line. (TIF) [file pone.0074388.s004.tif]
